# Long Term Variations of the Atmospheric Air Pollutants in Istanbul City

**DOI:** 10.3390/ijerph9030781

**Published:** 2012-03-05

**Authors:** H. Kurtulus Ozcan

**Affiliations:** Istanbul University, Engineering Faculty, Department of Environmental Engineering, 34320 Avcilar, Istanbul, Turkey; Email: hkozcan@istanbul.edu.tr; Tel.: +90-212-4737070-17726; Fax: +90-212-4737180

**Keywords:** air pollution, sulfur dioxide, nitrogen oxide, carbon monoxide, Istanbul city

## Abstract

High population density and intense industrial activity has resulted in various forms of pollution in megacities. Air pollution ranks at the top of this list. This study investigated long-term changes in air pollutant parameters (SO_2_, CO, NO, NO_2_, NO_x_) in Istanbul City, Turkey, using data from air-quality measurement stations on the Asian and European sides of Istanbul. The results show decreases from 2002 to 2010 in the amounts of SO_2_ (one of the main pollutants released as a result of the burning of fossil fuels) and CO (indicative of incomplete combustion). However, NO_x_ concentrations showed fluctuations over time, rather than a steady decline throughout the study period.

## 1. Introduction

Air pollution is one of the most important environmental problems, and concentrates mostly in cities. Atmospheric pollution is generally associated with by human activity and depends on economic development. Rapid urbanization may cause a range of environmental problems, such as air pollution, acid rain, water pollution and land pollution [1] (*i.e.*, solid wastes, toxic wastes, deforestation). Such environmental problems are much greater in the cities of developing countries, due to the overwhelming scale and speed of urbanization [2].

In both developed and rapidly industrializing countries, the major air pollution problem has typically been high levels of smoke and sulfur dioxide (SO_2_) arising from the combustion of sulfur-containing fossil fuels such as coal for domestic and industrial purposes [3,4]. Amongst the gaseous pollutants of atmospheric interest, SO_2_ is of major concern as a primary precursor to global acidification [5]. SO_2_ reacts on the surface of a variety of airborne solid particles, is soluble in water and can be oxidized within airborne water droplets, producing sulfuric acid. This acidic pollution can be transported by wind over many hundreds of kilometers, and is deposited as acid rain. Changes in the abundance of SO_2_ has an impact on atmospheric chemistry and on the radiation field, and hence on climate. Consequently, global observations of SO_2_ are important for atmospheric and climate research.

Transportation and road traffic is an important source of air pollution worldwide. Motor vehicles emit a wide variety of air pollutants, particularly carbon monoxide (CO), oxides of nitrogen (NO_x_), volatile organic compounds (VOCs) and particulate matter (PM), which have an increasing effect on urban air quality [6]. Carbon monoxide (CO) is a colorless and odorless gas that is formed during incomplete combustion of fossil fuels and is one of the major components of air pollution caused by traffic exhaust fumes. Formation of CO by vehicles depends on various factors including fuel formulation, engine conditions, air/fuel ratio, ignition timing, compression ratio and engine maintenance [7]. NO_x_concentrations are often used as an indicator of road traffic emissions at monitoring sites located in urban areas [6]. For energy production, coal is usually burnt in air and, therefore, atmospheric nitrogen can form NO. Moreover, organically-bound heterocyclic nitrogen compounds in coal are oxidized to NO during combustion and the sulfur in the coal is also oxidized to form sulfur dioxide. In the presence of oxygen, NO is easily transformed to NO_2_ and, for this reason, the mixture NO_2_ + NO is generally designated as NO_x_ [8]. In addition, photochemical reactions resulting from the action of sunlight on nitrogen dioxide (NO_2_) and VOCs from vehicles leads to the formation of ozone, a secondary long-range pollutant, that rural areas, often far from the original emission site. Acid rain is another long-range pollutant influenced by vehicle NO_x_ emissions [9,10].

Of the two constituents of NO_x_, NO_2_ is the more important for air quality since is more relevant for human health. It is an irritant gas, which has both chronic and acute effects and it is associated with respiratory and cardiovascular diseases [11,12]. Considering the role of NO_x_ as a precursor of a number of toxic pollutants and the effects that both short- and long-term exposure to NO_2_ concentrations can induce in it is clear that keeping atmospheric NO_2_ concentrations at low levels will provide significant public health benefits [13].

Many studies have examined the concentrations of air pollutants in cities [5,14,15,16]. Some studies also focus on traffic-generated air pollution in megacities [17,18,19,20]. Istanbul city is one of the fastest-growing cities in the world. There is intense concentration of both population and industrial facilities within Istanbul, and so several studies in the literature have reported on air pollution problems in the city [21,22,23,24]. This study investigated long-term changes in air pollutant parameters (SO_2_, CO, NO, NO_2_, NO_x_) in Istanbul, Turkey. Changes in air pollution levels in the city were determined using data obtained from air-quality measurement stations on the Asian and European sides of Istanbul.

## 2. Methodology

**Figure 1:**
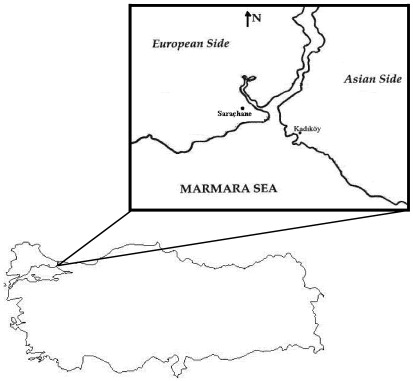
Study area and the air pollution stations.

Istanbul is located in northwestern Turkey within the Marmara Region. The Bosphorus, which connects the Sea of Marmara to the Black Sea, divides the city into a European side, comprising the historic and economic centers, and an Asian, Anatolian side (Figure 1). Apart from being the largest city and former political capital of the country, Istanbul has always been the centre of Turkey’s economic life because of its location at the junction of international land- and sea-trade routes. According to the Turkish Statistical Institute, Istanbul is the most crowded city in Turkey with a population of 13.6 million [25]. Istanbul is also Turkey’s largest industrial centre; it employs approximately 20% of Turkey’s industrial labor and contributes 38% of Turkey’s industrial workspace.

High population and intense industrial activity have resulted in various kinds of pollution in Istanbul. Air pollution ranks at the top of this list. This study investigated variation in air pollutant parameters (SO_2_, CO, NO, NO_2_, NO_x_), using data obtained from air pollution monitoring stations on both sides of Istanbul, for the period between January 2002 and December 2010. The data for the European side were obtained from the Sarachane measurement station and the data for the Asian side were obtained from the Kadikoy measurement station (Figure 1). Pollutant data were recorded in these stations and obtained from Istanbul Metropolitan Municipality Directorate of Environmental Protection department. Air pollutant was measured using the UV fluorescence principle for SO_2_, chemiluminescence principle for NO-NO_2_ and infrared absorption analysis for CO in ambient air.

## 3. Results and Discussion

**Figure 2:**
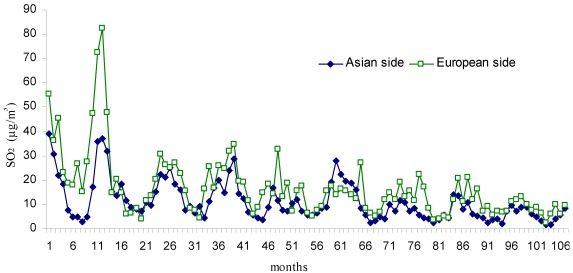
Monthly changes of SO_2_ (µg/m^3^) during the study period (Jan 2002–Dec 2010).

**Figure 3:**
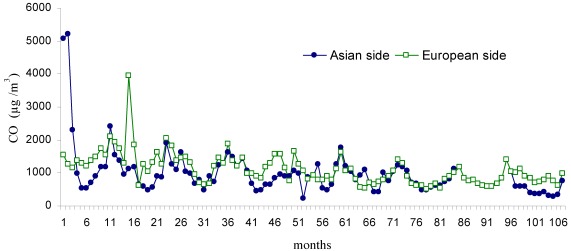
Monthly changes of CO (µg/m^3^) during the study period (Jan 2002–Dec 2010).

**Figure 4:**
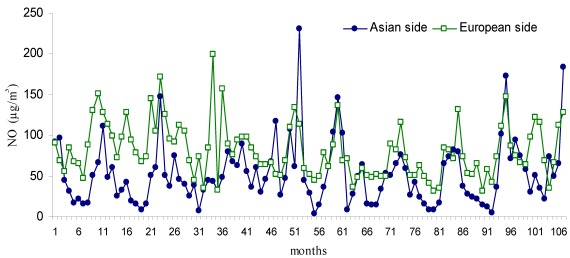
Monthly changes of NO (µg/m^3^) during the study period (Jan 2002–Dec 2010).

**Figure 5:**
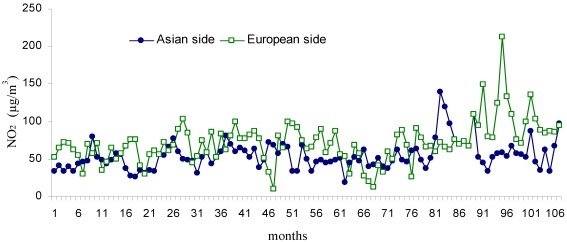
Monthly changes of NO_2_ (µg/m^3^) during the study period (Jan 2002–Dec 2010).

**Figure 6:**
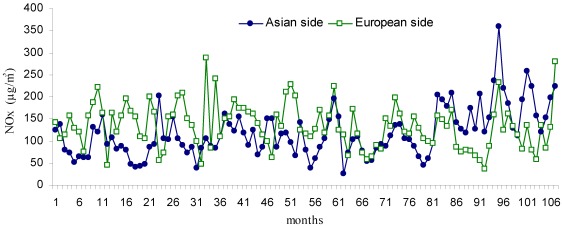
Monthly changes of NO_x_ (µg/m^3^) during the study period (Jan 2002–Dec 2010).

The mean monthly changes in air pollutant parameters (SO_2_, CO, NO, NO_2_ and NO_x_) for the period 2002–2010 are displayed in Figure 2, Figure 3, Figure 4, Figure 5, Figure 6, respectively. The seasonal changes in SO_2_ are clearly shown in Figure 2. The level of SO_2_ present in the city atmosphere was observed to increase during the winter months and decrease during summer. This is thought to result from the burning of fossil fuels for heating during winter. The mean concentration of SO_2_ throughout the study was determined as 16.6 µg/m^3^on the European side of the city and as 10.9 µg/m^3^ on the Asian side. The variations in CO concentration displayed similarities to those of SO_2_ concentration (Figure 3). The mean concentration for CO on the European side was 1103.4 µg/m^3^, compared with 963.6 µg/m^3^ on the Asian side. The highest CO concentrations were measured in March 2003 (month 15 of Figure 3) on the European side, and in January 2002 on the Asian side (month 1 of Figure 3).

The temporal changes in NO, NO_2_ and NO_x_ compounds are shown in Figure 4, Figure 5 and Figure 6. The mean NO concentration was 81.2 µg/m^3^ on the European side and 52.1 µg/m^3^ on the Asian side. The highest NO concentration for the European side was 199.86 µg/m^3^, measured in November 2004 (month 34 of Figure 4), compared with 231 µg/m^3^ in May 2006 (month 52 of Figure 4) for the Asian side. The mean concentration of NO_2_ was 71.4 µg/m^3^ on the European side and 54.2 µg/m^3^ on the Asian side. The highest NO_2_ concentration on the European side was 212.12 µg/m^3^ in December 2005 (month 95 of Figure 5) and 139.1 µg/m^3^ in November 2004 (month 82 of Figure 5) on the Asian side.

**Figure 7:**
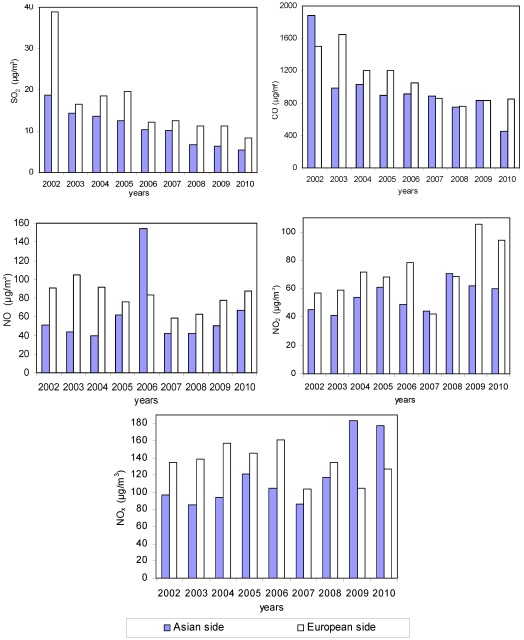
Yearly changes of air pollution parameters in Istanbul City.

The mean concentration of NO_x_ throughout the study was 134 µg/m^3^ on the European side and 117.7 µg/m^3^ on the Asian side. The highest NO_x_ concentration on the European side was 287.42 µg/m^3^in September 2005 (month 33 of Figure 6) and 359.46 µg/m^3^ in December 2009 (month 95 of Figure 6) on the Asian side. The annual changes in pollutant concentrations are presented in Figure 7. 

Plots of the changes in NO_x_ concentrations indicated that the NO_x_ concentration was higher during the winter months than in the spring and summer months. This difference is attributed to reasons such as not burning fossil fuels for heating during the summer months and the presence of adverse meteorological conditions during the winter months, which affect the air pollutant concentrations present. In addition, levels of NO_x_ compounds are reduced in summer as they are consumed in photochemical reactions, resulting in O_3_.

Figure 7 indicates that the levels of SO_2_ (one of the main pollutants released by burning fossil fuels) and CO (resulting from incomplete combustion) decreased from 2002 to 2010. One reason for this decline may be the widespread adoption of natural gas in Istanbul, beginning in the new millennium. Another factor is the increase in the quality standards of the coal and other fossil fuels used in the city and the enforcement of regulations limiting the use of coal containing high levels of sulfur.

As seen in Figure 7, the mean SO_2_ concentration between 2002 and 2010 declined by 21% on the European side of Istanbul and 28% for the Asian side. The annual changes in CO concentration are similar to those of SO_2_. The annual mean concentrations of CO decreased from year 2004 (Figure 7). In contrast to this finding, the changes in NO_x_ compounds fluctuated during the study period, rather than showing the steady declining trend observed for SO_2_ and CO (Figure 7). This may be because NO_x_ compounds are not only released by sources of heating but also from traffic-based pollution.

The magnitude of air pollution is determined by national air quality standards. Accordingly, long- and short-term boundary values of SO_2_, CO, and NO_2_ were determined as given in Table 1. It should be noted that Turkish air quality standards determine the long- and short-term boundary values of the pollutants, the long-term boundary value (LBV) gives the maximum allowable annual arithmetic mean and the short-term boundary value (SBV) gives the maximum daily arithmetic mean. In Table 1, EPA standards are also given for comparison. Observed SO_2_ and NO_2_ values in this study are lower than the LBV and SBV levels stated in Table 1. One the other hand, CO values exceed the maximum permissible level in the national air quality standards (Table 1). Particularly in winter seasons, CO levels are significantly higher. The main reason for this finding is the burning of fossil fuels for heating.

**Table 1:** Ambient Air Quality Standards.

Pollutant	National (Turkish) Standards [26]	EPA Standards [27]
Sulphurdioxide (SO_2_)	150 µg/m^3^ (LBV) ^a^	80 µg/m^3^ (annual arithmetic mean)
	400 µg/m^3^ (SBV) ^b^	365 µg/m^3^ (24 h average)
Carbonmonoxide (CO)	10 mg/m^3^ (LBV)	10 mg/m^3^ (8 h average)
	30 mg/m^3^ (SBV)	40 mg/m^3^ (1 h average)
Nitrogendioxide (NO_2_)	100 µg/m^3^ (LBV)	100 µg/m^3^ (annual arithmetic mean)
	300 µg/m^3^ (SBV)	

^a^ LBV: Long Term Boundary value (maximum allowable annual arithmetic mean). ^b^ SBV: Short Term Boundary value (maximum daily arithmetic mean).

## 4. Conclusions

Metropolitan cities are environments where air pollutants may be present at elevated levels, and may therefore directly affect public health. Istanbul has been exposed to extensive air pollution as a result of increasing population due to rural exodus; unplanned development and uncontrolled use of fossil fuels. This study investigated the temporal changes in air pollutant emissions in Istanbul over the period 2002 to 2010. One of the highlights of the study findings is that SO_2_ and CO emissions have decreased considerably in recent years. SO_2_ emissions resulting from the burning of coal and liquid fuels can only be reduced by lowering the sulfur content of the fuels. The controlled use of high-sulfur coal and the adoption of natural gas have caused SO_2_ emissions to decrease in Istanbul.

In conclusion, it has been observed that pollutant concentrations were kept well below specified limits between the years 2002 to 2010. The air pollution is thought to be lowered even further especially during the winter months as a result of the simple precautions that would be taken. NO_x_ and SO_2_ emissions may be reduced through the use of non-nitrogen or non-nitrogen/non-sulfur-containing fuels; or through the adoption of flue-gas treatment units in industrial plants. CO emission occurs under conditions of insufficient oxygen or due to suboptimal air/fuel mixture. Increased CO emissions usually stem from insufficiently calibrated combustion units. Improved calibration and maintenance of combustion units may therefore help to control CO emissions.
